# Role of Next-Generation Sequencing in Excluding the Nosocomial Origin of a Case of Legionnaires’ Disease Integrating Environmental Surveillance and Clinical Diagnosis

**DOI:** 10.3390/microorganisms14020486

**Published:** 2026-02-17

**Authors:** Francesco Paglione, Cataldo Maria Mannavola, Marilena La Sorda, Maria Luisa Ricci, Maria Scaturro, Silvia Laura Bosello, Roberta Masnata, Francesca Romana Monzo, Sara Vincenti, Patrizia Laurenti, Maurizio Sanguinetti, Flavio De Maio

**Affiliations:** 1 Department of Basic Biotechnological Sciences, Intensive and Perioperative Clinics, Università Cattolica del Sacro Cuore, L.go F. Vito 1, 00168 Rome, Italy; francesco.paglione01@icatt.it (F.P.); cataldomaria.mannavola01@icatt.it (C.M.M.); maurizio.sanguinetti@unicatt.it (M.S.); 2 Department of Laboratory and Hematological Sciences, Fondazione Policlinico Universitario A. Gemelli IRCCS, L.go A. Gemelli 8, 00168 Rome, Italy; marilena.lasorda@policlinicogemelli.it (M.L.S.);; 3 Department of Infectious Diseases, Istituto Superiore di Sanità, Viale Regina Elena 299, 00161 Rome, Italy; marialuisa.ricci@iss.it (M.L.R.); maria.scaturro@iss.it (M.S.); 4 ESCMID Study Group for Legionella Infections (ESGLI), 4051 Basel, Switzerland; 5 Division of Rheumatology and Clinical Immunology, Fondazione Policlinico Universitario A. Gemelli, IRCCS, L.go A. Gemelli 8, 00168 Rome, Italy; silvialaura.bosello@policlinicogemelli.it (S.L.B.); roberta.masnata@guest.policlinicogemelli.it (R.M.); 6 Department of Geriatric and Orthopedic Sciences, Division of Rheumatology, Università Cattolica del Sacro Cuore, L.go F, Vito 1, 00168 Rome, Italy; 7 Microbiota Analysis & Microbial WGS Research Core Facility, GSTeP, Fondazione Policlinico Universitario A. Gemelli IRCCS, L.go A. Gemelli 8, 00168 Rome, Italy; 8 Institute of Hygiene, Università Cattolica del Sacro Cuore, Lgo F, Vito 1, 00168 Rome, Italy; sara.vincenti@policlinicogemelli.it (S.V.); patrizia.laurenti@policlinicogemelli.it (P.L.)

**Keywords:** *Legionella pneumophila*, environmental surveillance, WGS, molecular epidemiology

## Abstract

*Legionella pneumophila* (*Lp*) remains one of the major causes of community- and hospital-acquired pneumonia, yet its diagnosis and source attribution continue to pose significant challenges. Here, we describe the case of an immunocompromised patient who developed Legionnaires’ disease during hospitalization. Following activation of the hospital’s internal surveillance system, *Lp* and *Legionella anisa* (*L. anisa*) were recovered from multiple water distribution points using a simplified culture-based protocol. Whole-genome sequencing (WGS) demonstrated that all environmental isolates belonged to a single clonal strain, whereas the clinical isolate was genetically unrelated, thereby excluding the hospital water system as the source of infection. Although not implicated in the patient’s disease, the detection of both *Lp* and *L. anisa* within the plumbing system highlighted underlying structural contamination and the potential masking effect of non-*L. pneumophila* species during culture-based surveillance. These findings support the integration of conventional microbiological methods with high-resolution genomic tools to enhance surveillance accuracy, support outbreak investigations, and strengthen public health responses. Overall, this case underscores the value of WGS as a decisive tool for source attribution, including the robust exclusion of a suspected nosocomial source, in complex clinical and environmental scenarios.

## 1. Introduction

The *Legionella* genus includes 67 species and over 72 serogroups, only a subset of which are associated with human disease [1]. Aquatic environments represent the primary reservoir of these Gram-negative bacteria [2]. The clinical relevance of *Legionella* spp. became evident following the 1976 outbreak in Philadelphia, which established *Legionella pneumophila* (*Lp*) as a paradigm of environmentally acquired bacterial pneumonia [3,4]. Among the different species, *Lp* is the main causative agent of legionellosis, which includes two distinct clinical entities: “Pontiac fever”, a self-limiting flu-like disease without pulmonary involvement, and “Legionnaires’ disease”, a severe form of pneumonia that can evolve into multiorgan failure and may be acquired in community, travel or healthcare settings [3]. Human infection typically results from inhalation of contaminated aerosols generated mainly by showers of man-made water systems, cooling towers, spa pools, and fountains [5].

Within aquatic environments, *Lp* survives and replicates, parasitizing free protozoa [2]. Following aerosol inhalation, the bacterium infects alveolar macrophages, where it evades host immune responses and establishes a replicative niche [6]. Advanced age, smoking habits, chronic lung diseases and conditions associated with impaired immunity, including immunosuppressive therapies, substantially increase the risk of infection and severe disease, particularly in high-income countries [5].

Despite the clinical relevance of legionellosis, its laboratory diagnosis remains challenging. The extensive taxonomic diversity of the genus limits the sensitivity of commonly used diagnostic tools [5]. In particular, the widely used urinary antigen test, which detects primarily *Lp* serogroup 1, leads to underdiagnosis of infections caused by non-serogroup 1 strains or other *Legionella* species [7]. Culture on Buffered Charcoal Yeast Extract (BCYE) agar remains the diagnostic gold standard, as it allows species-level identification and typing studies useful to determine the source of infection; however, its sensitivity is limited by slow bacterial growth, competition from other microorganisms, and the fastidious nature of the organism [8]. Molecular diagnostic approaches, including nucleic acid amplification tests, offer increased sensitivity and faster turnaround times but do not allow strain-level comparison or epidemiological linkage between clinical and environmental isolates [9,10]. In this context, whole-genome sequencing (WGS) has emerged as a powerful tool for high-resolution molecular epidemiology, enabling precise source attribution during outbreak investigations and sporadic cases alike [9].

*Legionella*-related diseases represent a substantial public health burden in the European Union, where they account for the highest morbidity among waterborne infections [11]. Consequently, prevention and control strategies rely on rigorous application of the Water Safety Plan suggested by the European drinking water directive 2184/2020 for priority premises such as hospitals and timely investigation of suspected nosocomial cases [2,11]. Integrating environmental surveillance with genomic analysis is increasingly recognized as essential for accurate risk assessment and effective infection control, also from the perspective of medical–legal liability.

## 2. Materials and Methods

### 2.1. Cultivation of Legionella pneumophila Isolates

An aliquot (0.1 mL) of BAL specimen was inoculated onto BCYE (Oxoid, Basingstoke, Hampshire, UK) and Glycine Vancomycin Polymyxin Cycloheximide (GVPC; ThermoFisher, Waltham, MA, USA) agar plates, which were incubated at 37 °C in an aerobic, humid environment with 5% CO_2_ [12]. Environmental isolates were obtained according to the guidelines of the Italian Ministry of Health [13,14].

Water samples were filtered using a sterile bottle-top filter with a pore size of 0.22 μm (Guangzhou Jet Bio-Filtration Co., Ltd., Guangzhou, China), connected to a vacuum system. Subsequently, the filter membranes were separated from the bottle top using a sterile scalpel and put into a sterile 50 mL Falcon tube (Corning Incorporated, Corning, NY, USA) full of 10 mL filtered water from the same sample. Each sample was then vortexed for two minutes, and an aliquot of the suspension was plated on BCYE and GVPC agar.

Physical decontamination was carried out by incubating a first aliquot of the suspension (1 mL) at 50 °C for 30 min, which was then plated as previously described.

Finally, the suspension containing the membrane was sonicated at 40 kHz for 10 min in an ultrasonic bath (Bactosonic^®^, Bandelin, Berlin, Germany), using a power setting of 200 Weff. Following this step, an aliquot was plated as described.

**Figure 1:**
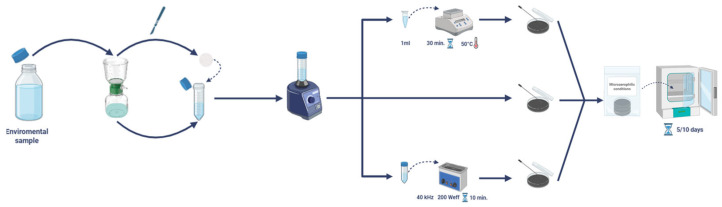
Workflow for the isolation of *Lp* from environmental samples.

All the plates were incubated at 37 °C under standard atmospheric conditions for 5–10 days and examined daily [13,14]. A schematic representation of the workflow is shown in Figure 1.

### 2.2. Monoclonal Antibody and Sequence-Based Typing

The serogroup determination of the *Lp* isolates was obtained using monoclonal antibodies provided by the Carl Gustav Carus University of Dresden, which also developed the Dresden monoclonal antibody (MAb)-typing scheme [15].

The sequence type was determined for both *Lp*1 and non-serogroup 1 *Lp* strains by sequence-based typing [16,17].

### 2.3. MALDI-TOF Identification

The positive samples were identified using the MALDI Biotyper^®^ (Bruker Daltonics, Bremen, Germany) system, operated with the MALDI Biotyper software MBT Compass 4.1 and the standard Bacterial Library (Bruker Daltonics, Bremen, Germany) under default parameter settings as recommended by the manufacturer; the bacterial colonies were picked up and spotted in duplicate onto the MALDI-TOF MS plate [18].

Spectra were acquired in a linear positive ion mode at a laser frequency of 60 Hz across a mass/charge ratio (*m*/*z*) of 2000 to 20,000 Da. Interpretation of results was performed according to the manufacturer’s instructions. The identification was considered highly probable with a log(score) ≥ 1.80, probable with a log(score) between 1.60 and 1.79, and not assigned with a log(score) < 1.60 [19].

Two GVPC agar plates were exclusively positive only for *Lp* (scores obtained: 2.37 and 2.36), whereas one BCYE agar plate yielded *L. anisa* (2.21).

All the positive samples were subcultured on BCYE agar plates and incubated at 37 °C under standard atmospheric conditions supplemented with 5% CO_2_ for 3–5 days [13,14]. After this time, the strains were cryopreserved in a 15% glycerol solution at −80 °C for subsequent analyses.

### 2.4. DNA Isolation, Whole Genome Sequencing and Analysis

Deep-frozen clinical strain and environmental isolates, stored in 15% glycerol solution at −80 °C, were thawed, plated on BCYE-agar plates, and incubated for 48 h. Single colonies were sub-cultured in Thioglycolate liquid medium (COPAN, Brescia, Italy) for 24 h before harvesting the bacterial culture. Total bacterial DNA was extracted using the DANAGENE Microbial DNA kit (Danagen-Bioted, Barcelona, Spain) [20]. Sequencing libraries were prepared using the Nextera DNA Prep library kit (Illumina, San Diego, CA, USA) for a 250 bp paired-end sequencing run (V2 cartridge) on an Illumina MiSeq instrument. (Illumina, San Diego, CA, USA) Sequencing depth was targeted to achieve a minimum coverage of 100× [21]. Run-quality metrics (Q30 and output) met the manufacturer’s specifications.

Raw data were analyzed by using a plug-in contained in the CLC genomics Workbecnh v.24.0.1 (QIAGEN, Hilden, Germany) [22]. FastQ files were quality-checked prior to adapter and quality trimming (quality limit: 0.05, corresponding to Phred score of 30; maximum number of ambiguous nucleotides: 2). Trimmed reads were de novo assembled by using default parameters (k-mer size: 20, bubble size 50 and minimum contig length of 500 bp). Read contamination was assessed using a k-mer-based algorithm, and coverage was determined by mapping original reads onto the obtained contigs. Average nucleotide identity (ANI) was calculated between isolates to reveal average genome similarity (setting a cutoff for *Legionella* subspecies of 96%); each assembly was finally annotated, searching for a set of contigs containing CDS with Pfam and GO terms. Each assembly was also used to search for antimicrobial resistance genes (AMRs) by running the DIAMOND plug-in with a database of peptide marker sequences. Plasmids’ and phages’ presence was investigated by using MOBscan v.3.1.9 and Phastest v.3.0, respectively [23,24]. In silico *Lp* sequence-based typing (SBT) was detected by using the legsta package v.0.5.1 [25].

Core genome multilocus sequence typing (cgMLST) was conducted using RidomSeqSphere (Ridom GmbH, Münster, Germany) [26], applying the *Lp* scheme described by Moran-Gilad et al., which included 1521 core genome loci [27]. Samples containing at least 95% of cgMLST targets were considered typeable. Alleles for each gene were assigned automatically by the RidomSeqSphere 11.1.2 software to ensure a unique nomenclature. The combination of all alleles in each strain formed an allelic profile that was used to generate minimum spanning trees (MSTs). Targets with missing values in one of the strains compared were omitted during distance calculation; each cluster was identified with a maximum of 4 single nucleotide variants (SNVs).

## 3. Results

### 3.1. Case Description

A 59-year-old male, active smoker, baker with a long-standing history of erosive and steroid-dependent rheumatoid arthritis (RA), was admitted in suboptimal clinical condition following a presentation to the emergency room (ER) with necrotic acral ulcers and significant tissue damage affecting the limbs. The patient reported an RA refractory to multiple lines of treatment: initially managed with Janus kinase (JAK) inhibitors and later switched to Abatacept. The patient did not show any rheumatoid nodules but presented multiple comorbidities, including type 2 diabetes mellitus, arterial hypertension, and glaucoma.

**Figure 2:**
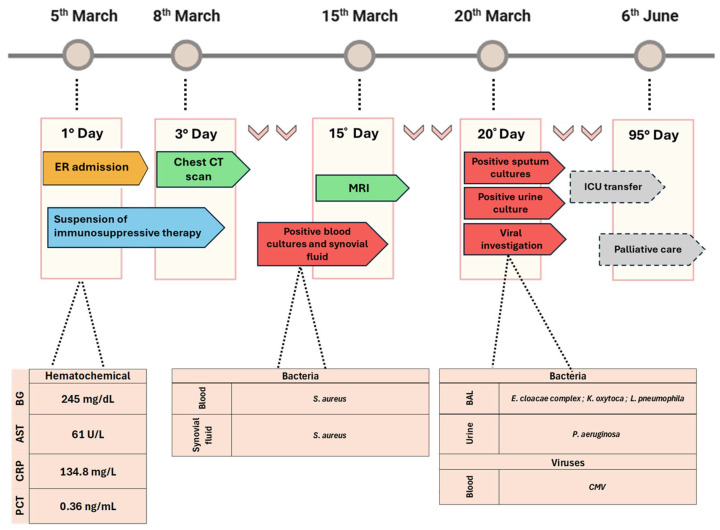
Schematic timeline reporting information on the clinical history of the patient. The figure reported the patient’s blood inflammatory markers (blood glucose levels (BG), aspartate aminotransferase (AST), C-reactive protein (CRP), procalcitonin (PCT), microbiological findings and diagnostic procedures during the hospitalization period.

As summarized in Figure 2, immunosuppressive therapy was suspended due to the substantial infectious risk upon admission. Laboratory investigations revealed inflammatory markers, including a C-reactive protein (CRP) concentration of 134.8 mg/L, aspartate aminotransferase (AST) mildly increased at 61 U/L, alkaline phosphatase levels at 138 U/L, and blood glucose levels (BG) were elevated at 245 mg/dL, while renal function was preserved with a creatinine level of 0.16 mg/dL. Procalcitonin (PCT) was slightly raised at 0.36 ng/mL. N-terminal proBNP (NT-proBNP) was significantly increased at 626 pg/mL (reference < 150 pg/mL), suggestive of cardiac strain. A positive lupus anticoagulant (LAC) and high titers of rheumatoid factor and anti-citrullinated protein antibodies (ACPA) were found (Figure 2).

Serial transthoracic echocardiograms ruled out endocarditis or other septic foci, while contrast-enhanced computed tomography (CT) revealed septic bursitis in the right shoulder, which was subsequently confirmed by magnetic resonance imaging (MRI). Additionally, the CT scan demonstrated early fibrotic changes, including interlobular septal thickening, bronchiectasis, and emphysematous changes, suggestive of pulmonary involvement.

On the tenth day of hospitalization, blood cultures were obtained due to clinical suspicion of an evolving infectious process; subsequent microbiological results revealed methicillin-sensitive *Staphylococcus aureus* in both blood cultures and synovial fluid (Figure 2), thereby prompting the initiation of targeted antimicrobial therapy with linezolid and cefazolin according to standard dosing regimens for MSSA infections.

Successive bronchoalveolar lavage (BAL) specimens revealed Gram-negative bacilli on direct microscopy. Aerobic cultures were positive for 10^5^ CFU/mL of the *Enterobacter cloacae* complex and *Klebsiella oxytoca*. Furthermore, *Lp* colonies were detected. Interestingly, the *Legionella* urinary antigen test was negative as well as the serological assays (IgM and IgG). Serogroup analysis revealed that the isolated strain belongs to serogroup 10. Given the concomitant radiological evidence of pneumonia and the isolation from a lower respiratory tract specimen, this finding was considered clinically relevant and not attributable to contamination, prompting *Legionella*-targeted antimicrobial therapy with cefepime and azithromycin to be started. Additionally, urine culture was positive for *Pseudomonas aeruginosa* at 10^6^ CFU/mL, correlating with the clinical suspicion of pyelonephritis, and viral investigations on peripheral blood specimens revealed cytomegalovirus (CMV) viremia, with a viral load of 2119 UI/mL (reference threshold > 122 UI/mL) (Figure 2).

Next laboratory examinations revealed a hyperproteinemia. Electroencephalography demonstrated diffuse slowing, and brain MRI indicated vasculitis, including T2-weighted/FLAIR hyperintensities and focal contrast enhancement. The suspicion of an immune-mediated encephalopathy prompted the administration of intravenous immunoglobulins and dexamethasone.

Recurrent episodes of desaturation and worsening conditions necessitated transfer to the intensive care unit (ICU); indeed, the next CT examination revealed consolidation in the pulmonary left lower lobe.

Despite intensive antimicrobial therapy and supportive care, the patient developed progressive respiratory deterioration characterized by extensive pulmonary consolidation, severe cachexia, and worsening gas exchange in the context of multiple concomitant pulmonary infections. Palliative care was therefore initiated, and the patient subsequently died.

### 3.2. Isolation of Legionella Strains from Environmental Samples and Genomic Characterization

**Figure 3:**
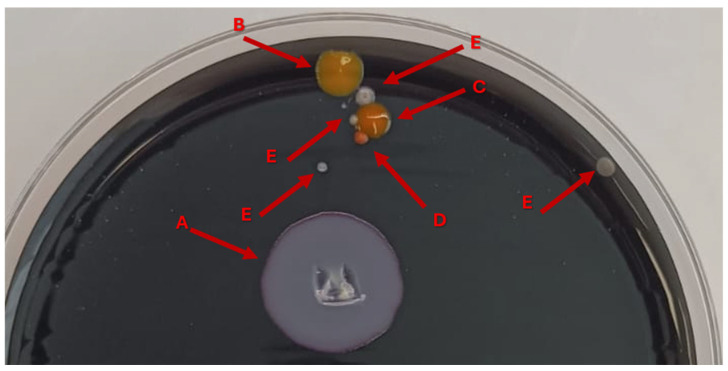
Colonies grow on GVPC selective agar from an environmental specimen. Morphologically distinct colonies were retrieved after 5 days of incubation at 37 °C. Red arrows indicate: (**A**) *Lp*; (**B**) *Brevundimonas diminuta*; (**C**) *Chryseobacterium shandongense*; (**D**) *Staphylococcus capitis*; and (**E**) *Staphylococcus warneri*.

Environmental sampling was performed at multiple water distribution points within the patient’s room (Table 1, Figure 3), as previously described [13,14].

As shown in Figure 1, *Lp* was isolated directly from the untreated sample (2406G0261) as well as from post-decontaminated (at 50 °C) and post-sonicated samples. Furthermore, *L. anisa* was isolated and identified from a hand shower bidet (2406G0262).

**Figure 4:**
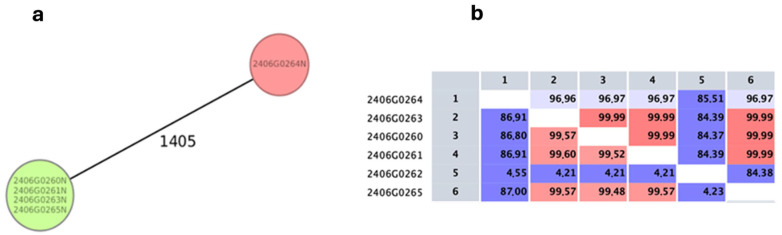
Genomic characterization of *Legionella* strains. (**a**) Minimum spanning tree based on cgMLST showing the genetic distance among the *Lp* clinical isolate (2406G0264) and environmental isolates (2406G0260, 2406G0261, 2406G0263, 2406G0265). (**b**) Pairwise average nucleotide identity (ANI) heatmap among the isolates (values expressed as %), including *L. anisa* as an outgroup. The clinical isolate shows low genomic similarity to the environmental cluster, supporting lack of relatedness.

Comparative genomic analysis revealed that the environmental *Lp* isolates (2406G0260, 2406G0261, 2406G0263, 2406G0265) formed a single clonal cluster, showing no allelic differences by core genome multilocus sequence typing (cgMLST) (Figure 4a) and exhibiting high average nucleotide identity (ANI) values (≥99.5%, Figure 4b). In contrast, the clinical isolate 2406G0264 was genetically unrelated to the environmental *Lp* strains, displaying a distinct MST profile and markedly lower ANI values with the other strains. Isolate 2406G0262 was molecularly confirmed to be *L. anisa* and showed ANI values were well below the species-level threshold, consistent with species-level divergence. No plasmids or prophages were detected in any isolate. While environmental isolates shared an identical resistome (9-Aminoglycoside phosphotransferase type Ia (APH(9)-Ia), providing intrinsic resistance to the spectinomycin), the clinical strain carried a different set of antibiotic resistance genes (LpeA and LpeB, two subunits of the LpeAB efflux pump, which provides macrolide resistance). Altogether, these findings indicate that the clinical isolate is not phylogenetically related to the environmental *Lp* strains. These genomic findings conclusively exclude a direct epidemiological link between the clinical isolate and the hospital water system.

## 4. Discussion

The genus *Legionella* comprises multiple species and serogroups, among which *Lp* is considered the most clinically relevant in human pathology as the primary etiological agent of Legionnaire’s disease [1]. The burden of disease is associated with *Legionella* spp. infections in many industrialized countries and remains the highest among waterborne diseases [28,29]. *Lp* naturally inhabits both natural and artificial aquatic environments, where it parasitizes free-living protozoa [2]. Consequently, human infection typically occurs through inhalation of contaminated aerosols generated from water systems, including those in healthcare settings [5]. Importantly, the challenge of controlling *Legionella* infection is still in its early stages and involves not only scientific and healthcare fields but also global health policies [30]. In a recent review, Hammes and colleagues established different points regarding *Legionella* infection control [2]. While there is a growing interest in the *Legionella* species diversity and cooccurrence and in the associated diagnostic test, beyond *Lp* only, the assessment of *Lp* environmental prevalence remains elusive and underestimated [2,31,32].

In this study, we describe a case of Legionnaires’ disease involving a multidisciplinary team that developed severe pneumonia following admission to the emergency department. Microbiological investigations revealed the presence of *Lp*, which is known to lack distinctive clinical features that allow it to be reliably differentiated from other forms of pneumonia; therefore, laboratory diagnosis must be considered an essential and pivotal component of the clinical diagnostic workflow. Accordingly, an environmental prevention and control surveillance protocol, based on the application of the Water Safety Plan, was initiated to assess the hospital water distribution system with the aim of investigating a suspected healthcare-associated scenario.

Diagnostic procedures for *Lp* detection remain challenging, as they are often time-consuming and affected by limited sensibility [30]. Although molecular assays, including PCR or rapid nucleic acid amplification test (NAAT)-based assays, can improve detection rates, they do not allow strain-level comparison between clinical and environmental isolates, limiting their value for source attribution [5].

In contrast, WGS provides high-resolution molecular data that enables precise phylogenetic analysis, making it particularly suitable for epidemiological investigations.

In the present study, WGS analysis demonstrated that environmental *Lp* isolates formed a single clonal cluster expression of an environmental contamination that needs to be monitored, whereas the clinical isolate was genetically unrelated, effectively excluding the hospital water system as the source of infection. Importantly, this negative genomic finding is clinically and operationally meaningful, as it prevents misclassification of the case as hospital-acquired and supports proportionate infection-control and medico-legal decision-making.

Culture-based detection of *Lp* remains a critical component of both clinical diagnosis and environmental surveillance, despite well-recognized limitations [30]. One major challenge is the viable but non-culturable (VBNC) state of *Lp*, in which bacteria remain alive, potentially pathogenic, but not recoverable using standard culture methods [33]. This phenomenon may lead to underestimation of environmental contamination, particularly in complex water systems [34]. In addition, infections caused by *Lp* serogroups other than serogroup 1 are frequently missed by urinary antigen testing, further complicating diagnosis [9]. In this context, the use of enrichment methods may improve both culture and molecular sensitivity [35]. Additionally, the organization of laboratory activities for *Legionella* diagnosis structured as a standardized diagnostic workflow and the use of rapid molecular tests may indirectly enhance *Lp* isolation by culture when applied to respiratory samples [20]. In this case, the clinical interpretation of *Lp* isolation is particularly complex. Although the organism was recovered from a BAL specimen in a patient with radiological evidence of pneumonia, the simultaneous presence of multiple pulmonary pathogens makes it difficult to classify the condition as classical “Legionnaires’ disease” caused by a single etiological agent. Instead, this case represents a multifactorial pulmonary infectious scenario in which *Lp* was a clinically relevant component among several co-infections.

Hence, a feasible, reproducible, and effective flowchart for the bacterial isolation from environmental samples is important [36]. While metagenomics may improve *Lp* diagnosis on clinical samples, only pathogen isolation may improve a deep genomic characterization to compare clinical and environmental isolates. Indeed, molecular epidemiology represents a valuable tool for microbiological surveillance and epidemiological programs and may potentially serve as incontrovertible legal evidence [37]. Indeed, just in Italy, *Lp* disease is responsible for 3.2% of hospital cases, with a case-fatality ratio of 56% with respect to 15,1% of community-acquired Legionnaires’ disease [38]. Although the proportion of hospital cases is relatively small compared to the total, the high mortality rate in patients who contract legionellosis in hospitals is very high [28,38].

Beyond its microbiological implications, accurate source attribution has important medico-legal and risk management consequences, particularly in healthcare settings. NGS-based procedures could therefore be integrated into risk management strategies under the guidance of expert microbiologists. A limitation of this study is the lack of environmental investigation of the patient’s domestic setting and workplace, which prevents conclusions regarding a possible source of infection. Nevertheless, the integration of environmental surveillance with WGS provided sufficient resolution to exclude the hospital water system as the origin of the infection.

In conclusion, this study supports the integration of whole-genome sequencing into routine environmental surveillance and epidemiological investigations of *Lp* infections. The implementation of a cost-effective and reproducible isolation method would improve pathogen recovery rate, and WGS should represent a second-line, high-resolution tool to be deployed in selected scenarios where strain/clone-level relatedness is required to confirm and/or to exclude suspected transmission pathways (e.g., suspected healthcare-associated cases, clusters/outbreak investigations, or medico-legal disputes), ideally through centralized reference laboratories in a hub-and-spoke model in addition to conventional standardized culture and molecular methods.

## Figures and Tables

**Table 1:** Samples analyzed and their origin.

Strain Code	Type	Specimen Origin	*Legionella* spp. Detected
2406G0260	Environmental	Shower (I sonication)	*Lp* *
2406G0261	Environmental	Shower (pure sample)	*Lp* *
2406G0262	Environmental	Hand shower bidet	*L. anisa* *
2406G0263	Environmental	Shower (II sonication)	*Lp* *
2406G0264	Clinical	Clinical isolate	*Lp*
2406G0265	Environmental	Boiler	*Lp*

*: Strain isolated by the protocol illustrated in Figure 1.

## Data Availability

Data may be available upon request to the corresponding author.
